# User satisfaction in the spanish health system: trend analysis

**DOI:** 10.11606/S1518-8787.2019053001506

**Published:** 2019-09-18

**Authors:** Víctor Pérez-Cantó, Loreto Maciá-Soler, Víctor M González-Chordá

**Affiliations:** I Universidad de Alicante. Departamento de Enfermería. Alicante, AL, España; II Univesidad de Alicante. Unidad Científica de Innovación Empresarial “Ars Innovatio”. Alicante, AL, España; III Universitat Jaume I. Departamento de Enfermería. Castellón, CS, España

**Keywords:** Patient Satisfaction, National Health Systems, Quality of Health Care, Health Services Research

## Abstract

**OBJECTIVE:**

To analyze the trend of opinion and satisfaction indicators of the Spanish national health system from 2005 to 2017.

**METHODS:**

Ecological study of time series analyzing the trend of eight indicators of opinion and satisfaction on the Spanish national health system and its autonomous communities from 2005 to 2017. The data was obtained from the Ministry of Health, Social Services and Equality and from the Health Barometer. The Prais-Winsten regression method was used.

**RESULTS:**

A static tendency was observed in the perception of users on how the health system works (APC = 1.898, 95%CI -0.954 – 4.751) and decreasing opinion on the improvement of primary care (APC = -0.283; 95%CI -0.335 – -0.121), specialized (APC = -0.241, 95%CI -0.74 – -0.109) and hospitalization (APC = -0.171, 95%CI -0.307 – -0.036). Satisfaction with knowledge and follow-up by the family doctor and pediatrician showed an increasing trend (APC = 7.939, 95%CI 3.965 – 11.914). Satisfaction with medical and nursing professionals was static. No large differences were observed in the trends of the indicators studied in the autonomous communities.

**CONCLUSIONS:**

A negative trend was observed in the opinion of the Spanish national health system users. Financing, human resources, quality management systems and differences in the autonomous communities may be some of the causes.

## INTRODUCTION

Health systems aim to improve the health of citizens through healing, prevention and rehabilitation. However, they are influenced by political, social, cultural and economic factors of each country. Quality management systems ensure the intrinsic and noticed quality of the services. The intrinsic quality is focused on the design, execution and assessment of processes. The noticed quality aims at the evaluation and satisfaction of the users^1^ .

Satisfaction, understood as the ability to generate a positive experience for users and the population in contact with health services, has been widely studied since the 1960s despite its subjective nature^2^ . Governments and policy analysts have used patient satisfaction as an approach to assess the performance of health systems. The institutions regularly monitor the satisfaction of their patients and develop strategies to improve quality and achieve a better position in the market. The information about satisfaction allows us to predict, among other things, therapeutic compliance and the possible return before a new episode. The two strategic points and their result directly influences the costs, profitability and sustainability of the organizations^3^ .

The evaluation of user satisfaction is not exempt from methodological problems. Satisfaction is influenced by patient characteristics such as age, gender, marital status, education, income level or health status^4 , 5^ . Older people are usually more satisfied, and the dissatisfaction increases as their health worsen^6^ . In addition, custom-made measuring instruments are commonly used, validated in a few cases^7^ .

The influence of socioeconomic factors should also be considered. The recent economic crisis in Europe has put great pressure on health systems. Some countries took restrictive measures such as reducing the service portfolio and expenses on medication or cutting personnel and salaries. These measures can increase social inequalities in healthcare coverage and have a negative impact on the safety and satisfaction of users^8^ .

There are ways to control spending and improve profitability based on the implementation of quality management systems and on the improvement of care outcomes, for example, hospital mortality, reduction of stays, readmissions or patient satisfaction, among others. Therefore, patient satisfaction has been established as a key indicator of outcome to evaluate the quality and efficiency of health services, coinciding with the strategic lines of the World Health Organization (WHO)^9^ .

The national health system is launched in Spain, 1986, after the enactment of the General Health Law^10^ . Some main characteristics are the right of every citizen to health, public financing and provision of services that guarantee the quality of care, with decentralized health system in autonomous communities.

The process of decentralization of competencies ended in 2001. In 2003, the Law of Cohesion and Quality of the National Health System was enacted to guarantee citizen participation, quality and equity of assistance in the national territory. This law establishes the fundamental principles on quality of care and the development of tools such as the portfolio of services, training and development of professionals, research, unique digital clinical history, use of guides, protocols and indicators, or a single information system^11^ . However, it does not specify the need to establish a quality management system for the entire national territory or in its autonomous communities. After 15 years since its implementation, some of the main problems are: lack of equity in spending and financing, the health level of citizens, accessibility to health services and use of resources^12^ .

Providing information related to quality is an aspect of public health interest. Improving health systems and, therefore, examining trends in user satisfaction can make it possible to evaluate the impact of the health policies adopted, observing the influence of these changes on the opinions, experiences and attitudes of citizens regarding health care^13^ . Trend studies in Spain are limited and there is no trend analysis of indicators related to the satisfaction of the healthcare system users.

This study aimed to analyze the trend of the opinion and satisfaction indicators of the Spanish National Health System between 2005 and 2017.

## METHODS

Ecological study of time series that analyzed the satisfaction trend of users of the Spanish national health system and its autonomous communities, between 2005 and 2017.

The variables were satisfaction with how the health system works, with information and with the care and attention received, in addition to the improvement noticed by the users. For this, the trend of eight indicators of opinion and user satisfaction on the Spanish national health system and its autonomous communities was analyzed. Three indicators were obtained from the series of Key Indicators of the National Health System (NHS) and its autonomous communities, through the Health Information System of the Ministry of Health, Social Services and Equality^14^ , and five indicators of the Health Barometer^15^ ( Table ).

**Table:** Satisfaction indicators selected to carry out the study. Spain, 2018.

Indicator	Formula/question
Key Indicators	

Satisfaction with the operation of the NHS^a^	Ratings average of the satisfaction degree collected on a *Likert* scale of 1 (“very dissatisfied”) to 10 (“totally satisfied”).
Satisfaction with the knowledge of the clinical history and the monitoring of their health problems by the family doctor and the pediatrician^b^	
Satisfaction with the information received in the specialist doctor’s appointment^a^	

Health barometer	

Satisfaction with care and attention by medical staff^c^	Ratings average of the satisfaction degree collected on a *Likert* scale of 1 (“totally unsatisfactory”) to 10 (“totally satisfactory”).
Satisfaction with the care and attention by the Nursing staff^c^	
Percentage of respondents who believe that primary care has improved^c^	In your opinion, has each of the following health care services improved, worsened or remains the same over the last five years?
Percentage of respondents who believe that specialized care consultations have improved^c^	
Percentage of respondents who believe that hospitalization has improved^c^	

NHS: National Health System

^a^ Last year of available data: 2017.

^b^ Last year of available data: 2013.

^c^ Last year of available data: 2016.

The series of Key Indicators make it possible to observe changes and trends since 1990. Currently, the complete list amounts to 247 indicators (accessibility, health status, resources, spending, security and satisfaction, among others). Indicators with standardized definitions and international use are studied, which are accessible for consultation and analysis in a single repository. These indicators are calculated by obtaining data from different sources with broad population coverage, such as the Minimum Basic Data Set for hospital discharges, the Primary Care Information System, intercensal statistics from the National Institute of Statistics or the National Health Survey, among others^14^ .

The Health Barometer is an opinion study, conducted annually, carried out since 1993 by the same ministry in collaboration with the Spanish Sociological Research Center. It is designed to know the degree of citizen satisfaction with public health services, the impact of measures linked to health policies, the level of knowledge of citizens and public opinion on these policies. The information is collected through questionnaires, with a multi-stage sample design, stratified by conglomerates, with random selection of the sampling units, and a total of 7,800 annual surveys^15^ .

According to the definitions of the indicators in the NHS Information System (Key Indicator) or the Health Barometer, the indicators included in this study are estimated by means, adding the satisfaction degree assessments, divided by number of individuals surveyed, except for the last three indicators that are estimated as percentages.

Data have been included since 2005, because in previous years the series were interrupted, and data were not available in the indicators analyzed. The last year was 2017, except for an indication that the historical series ended in 2013 or 2016, according to the availability of data from the health information system. The autonomous cities of Ceuta and Melilla were excluded, since the complete historical series were not available.

The estimation of the trends was based on the calculation of the annual percentage change (Annual Percent Change – APC) and its 95% confidence intervals (95%CI). The Praes-Wisten regression method was used for the analysis of time series of quantitative data. Thus, a global trend was obtained for a certain period and was determined as increasing (95%CI is positive and does not include the value 0), static (95%CI includes value 0), or decreasing (95%CI is negative and does not include the value 0). The analysis was performed using the Stata 14.0 program.

The study was carried out with aggregate data obtained from public sources, being openly accessible for consultation and exploitation. For this reason, the approval of the study was not requested by an ethics and research committee.

## RESULTS

The degree of satisfaction with how the health system works increased from 6.25 in 2005 to 6.68 in 2017, showing a static trend in all the communities and in the whole country, except for Madrid, that showed an increasing trend (APC = 7.451; 95%CI: 0.433–14.470). Asturias was the only place that showed a negative annual variation, although it was not significant ( Table 1 ). The information received in the specialist physician appointment was static in all Spain (APC = 2.585, 95%CI -2.078–2.085) and in the autonomous communities, except for the Canary Islands with a significant increasing trend (APC = 0.942, 95%CI -0.200–6.337). There was a negative non-significant annual variation in the Baleares, Asturias, Rioja, Extremadura, and Cataluña.

**Table 1:** Degree of satisfaction with how the public health system works. Spain, 2018

CCAA	2005	2017	APC	95%CI	Trend
Andalucía	6.14	6.27	1.031	-4.239–6.302	Static
Aragón	6.41	7.29	2.210	-2.114–6.536	Static
Asturias	7.26	7.22	-0.239	-3.338–2.859	Static
Baleares	5.83	6.98	0.845	-2.560–4.252	Static
Canary Islands	5.37	6.10	1.014	-1.749–3.778	Static
Cantabria	6.33	7.35	1.542	-1.884–4.969	Static
Castilla and León	6.40	7.07	0.735	-1.677–3.148	Static
Cataluña	5.96	6.51	1.012	-3.033–5.058	Static
Extremadura	6.30	6.61	0.726	-2.176–3.629	Static
Galicia	5.56	6.49	2.302	-0.922–5.527	Static
La Mancha	6.61	6.65	0.388	-2.036–2.813	Static
Madrid	6.10	6.82	7.451	0.433–14.470	Increasing
Murcia	6.01	7.10	1.475	-1.455–4.405	Static
Navarra	6.71	7.06	1.246	-2.453–4.946	Static
Basque Country	6.51	7.33	1.265	-2.348–4.879	Static
Rioja	6.56	6.99	0.090	-4.924–5.106	Static
Valencia	6.12	6.71	1.852	-2.340–6.045	Static
Spain	6.25	6.68	1.898	-0.954–4.751	Static

CCAA: autonomous communities; APC: annual percent change

The trend in satisfaction with the knowledge of the clinical history and follow-up by the family doctor and pediatrician was increasing in all Spain (APC = 7.939, 95%CI 3.965–11.914), increasing by 0.6 points in the period. Madrid, Andalucía, Navarra, Canarias and Galicia showed a significant increasing trend. In the rest of the autonomous communities, the trend was static. The communities that showed a decreasing annual variation were Baleares and Asturias ( Table 2 ).

**Table 2:** Degree of satisfaction with the information received in the specialist and family doctor appointment about their health. Spain, 2018

CCAA		2005	2017*	APC	95%CI	Trend
Andalucía	Specialist	7.26	7.12	0.364	-5.344–5.250	Static
	Physician	6.97	7.59	6.183	2.799–9.567	Increasing
Aragón	Specialist	7.16	7.71	1.248	-3.314–3.403	Static
	Physician	7.82	8.18	1.441	-1.953–4.835	Static
Asturias	Specialist	8.02	7.24	-0.881	-1.622–4.118	Static
	Physician	7.44	7.31	-0.034	-2.638–2.568	Static
Baleares	Specialist	7.32	6.8	-2.711	-4.044–4.773	Static
	Physician	7.00	7.29	-0.702	-3.226–1.821	Static
Canary Islands	Specialist	6.32	6.84	0.942	0.200–6.337	Increasing
	Physician	6.87	7.42	5.202	2.236–8.168	Increasing
Cantabria	Specialist	7.22	7.65	1.479	-3.484–4.951	Static
	Physician	7.63	7.76	0.555	-3.261–4.372	Static
Castilla and León	Specialist	7.12	7.35	0.423	-2.745–6.207	Static
	Physician	7.24	7.65	1.504	-3.262–6.272	Static
Cataluña	Specialist	6.87	6.95	-0.046	-2.255–5.214	Static
	Physician	6.67	7.4	2.968	-2.179–8.117	Static
Extremadura	Specialist	7.59	7.56	-0.273	-6.633–1.210	Static
	Physician	7.53	7.46	2.044	-4.223–8.313	Static
Galicia	Specialist	6.08	7.14	2.879	-1.463–1.144	Static
	Physician	6.46	7.33	4.063	0.837–7.289	Increasing
La Mancha	Specialist	7.17	7.48	0.888	-2.465–4.192	Static
	Physician	7.30	7.96	3.199	-0.920–7.319	Static
Madrid	Specialist	6.68	7.08	0.863	-0.945–6.705	Static
	Physician	7.03	7.47	8.959	5.640–12.279	Increasing
Murcia	Specialist	7.06	8.07	3.268	-4.695–9.865	Static
	Physician	7.15	7.62	1.094	-3.678–5.868	Static
Navarra	Specialist	7.09	7.42	0.044	-2.796–4.572	Static
	Physician	7.00	8.29	5.771	4.948–6.594	Increasing
Basque Country	Specialist	6.86	7.21	0.733	-5.287–4.740	Static
	Physician	7.03	7.55	0.587	-2.971–4.146	Static
Rioja	Specialist	7.64	7.2	-0.477	-3.507–1.744	Static
	Physician	7.54	8.04	1.278	-1.923–4.481	Static
Valencia	Specialist	6.57	7.26	1.730	-3.150–2.195	Static
	Physician	6.67	7.46	2.637	-1.903–7.178	Static
Spain	Specialist	6.91	7.19	2.585	-2.078–2.085	Static
	Physician	6.96	7.56	7.939	3.965–11.914	Increasing

CCAA: autonomous communities; APC: annual percent change

* The data related to the satisfaction of the family doctor are from 2013.

The satisfaction of the users with the nursing staff showed a static trend in all Spain (APC = 1.898, 95%CI -0.954–4.751) and in all the autonomous communities. Similarly, the attention by the medical staff was static (APC = 3.227, 95%CI -7.076–13.531), as were in all the autonomous communities, except for Asturias, which showed a decreasing trend (APC = -10.486; 95%CI -12.798 – -8.174) with a decrease of more than 10 points ( Table 3 ).

**Table 3:** Degree of satisfaction with the care received by nursing and medicine professionals. Spain. 2018.

CCAA		2005	2016	APC	95%CI	Trend
Andalucía	Nursing	7.51	7.28	0.108	-4.407–4.625	Static
	Medicine	7.46	7.15	-0.110	-4.374–4.153	Static
Aragón	Nursing	7.85	8.16	1.563	-3.377–6.504	Static
	Medicine	7.86	8.25	1.250	-2.501–5.002	Static
Asturias	Nursing	8.25	7.49	-1.069	-3.682–1.543	Static
	Medicine	8.19	7.36	-10.486	-12.798– -8.174	Decreasing
Baleares	Nursing	7.29	7.82	0.036	-3.197–3.270	Static
	Medicine	7.15	7.52	-0.296	-3.447–2.853	Static
Canary Islands	Nursing	6.90	7.57	0.771	-3.325–4.868	Static
	Medicine	6.70	7.36	1.162	-3.327–5.652	Static
Cantabria	Nursing	7.60	8.04	1.690	-4.130–7.510	Static
	Medicine	7.61	7.95	0.857	-3.040–4.755	Static
Castilla and León	Nursing	7.08	7.40	1.939	-3.715–7.593	Static
	Medicine	7.47	7.55	1.380	-4.459–7.219	Static
Cataluña	Nursing	7.39	7.46	1.219	-4.562–7.001	Static
	Medicine	7.24	7.23	0.288	-4.814–5.391	Static
Extremadura	Nursing	7.66	7.53	0.415	-3.552–4.383	Static
	Medicine	7.60	7.53	0.151	-3.989–4.291	Static
Galicia	Nursing	6.39	7.28	1.279	-1.939–4.499	Static
	Medicine	6.28	7.13	1.252	-2.262–4.768	Static
La Mancha	Nursing	7.56	7.68	0.142	-3.511–3.797	Static
	Medicine	7.18	7.22	-0.306	-3.996–3.384	Static
Madrid	Nursing	7.14	7.58	2.342	-3.964–8.650	Static
	Medicine	6.98	7.48	1.251	-3.216–5.719	Static
Murcia	Nursing	6.96	7.47	1.505	-2.687–5.698	Static
	Medicine	6.92	7.66	2.699	-1.658–7.058	Static
Navarra	Nursing	7.67	8.00	0.816	-2.933–4.565	Static
	Medicine	7.55	7.85	0.625	-2.668–3.918	Static
Basque Country	Nursing	7.36	7.87	4.188	-1.835–10.218	Static
	Medicine	7.14	7.65	3.531	-2.631–9.694	Static
Rioja	Nursing	8.16	7.75	-0.458	-3.543–2.626	Static
	Medicine	7.93	7.75	0.006	-3.867–3.879	Static
Valencia	Nursing	7.02	7.20	0.654	-5.079–6.389	Static
	Medicine	6.96	7.25	1.127	-4.766–7.021	Static
Spain	Nursing	7.30	7.48	1.898	-0.954–4.751	Static
	Medicine	7.20	7.37	3.227	-7.076–13.531	Static

CCAA: autonomous communities; APC: annual percent change

We observed a decreasing trend in the improvement perceived by users in primary care services in all Spain (APC = -0.238, 95%CI -0.335 – -0.121), as well as in five communities (Asturias, Cantabria, Andalucía, Cataluña and Madrid). The percentage of users who believe that the attention in specialized consultations has improved showed a decreasing trend in Spain (APC = -0.241, 95%CI -0.74 – -0.109) and four communities (Basque Country, Asturias, Andalucía and Cantabria); the others were static. Likewise, a decreasing trend was observed in hospitalization services in Spain (APC = -0.171, 95%CI -0.307 – -0.036) and in three autonomous communities (Murcia, Asturias and Andalucía). No community showed an increasing trend in the percentage of respondents who assessed whether the attention in these three services had improved in the last five years.

## DISCUSSION

In general, there was a static trend in Spain and most of its autonomous communities in the indicators: degree of satisfaction with how the public health system works, information received by the specialist doctor and knowledge of clinical history, as well as follow-up by the family physician and pediatrician. There are few studies of trends in satisfaction at national and international levels and there is little research that links trends to improvements made based on patient satisfaction^16^ . However, the cases with an increasing tendency on the indicators linked to the development of the digital clinical history of the NHS in Spain are related to improvements identified by the users when they go to a health center looking for information about their illness and the order in their documentation^17^ .

Analysts argue that more financing does not necessarily lead to better satisfaction and cannot explain a large part of the difference in satisfaction ratings between countries^18 , 19^ . A study conducted in France^18^ with more than 10,000 patients showed that they were satisfied with their hospital stay despite reductions in hospital costs. In Spain, an analysis of trends on specialized care indicators^19^ showed an increase of 9.5 points on spending, with a decreasing trend of users’ satisfaction for these services and worse health outcomes (increase of infections and in-hospital mortality).

The latest annual report of the Spanish national health system^20^ shows how some autonomous communities with high healthcare costs (Basque Country, Extremadura, Navarra, Murcia, Asturias and Cantabria) presented positive ratings of users in most of the indicators, while Baleares and Andalucía showed worse results in most indicators, coinciding with lower health expenses. The heterogeneity of the autonomous communities in financing can influence the results of user opinion. In addition, a north-south gradient was observed, since the best results in the indicators studied were in the northern half of the country and the worst in the south. This gradient has already been mentioned in previous research^21^ , related to the decline in public spending on health and an increase in inequalities between autonomous communities.

A decreasing trend was observed in the percentage of patients who believe that primary care, specialized care consultations and hospitalization, three pillars of the health system, have improved. The hospitalization results coincide with other trend studies in France^18^ and Germany^22^ . This suggests that aspects related to quality such as information, comfort and agility of systems increasingly influence the satisfaction of users^23^ .The main problems in primary care may be underfunding and the heterogeneity caused by the decentralization of competencies^24^ . However, the possible strategies to be implemented must be well-thought. A study conducted in Finland between 1998 and 2011 showed that, despite government reforms, patients were less satisfied with primary care, especially with accessibility and continuity of care^25^ .

On the other hand, satisfaction with nursing professionals and physicians in all Spain showed a static trend, despite the increasing trend of nursing and medical professionals in recent years^19^ . Autonomous communities with a high degree of satisfaction in relation to nursing and medical professionals had the highest rates of professionals^20^ , observing the same north-south gradient. The influence of the rate of professionals on the quality of care and satisfaction is addressed by various authors, although no studies have been identified at national level related to medicine or nursing.

Spain has a medical rate of 3.8 for every thousand inhabitants in its NHS (the European average is 3.5), ranking seventh among the countries with more doctors in Europe. The rate of nurses in Spain is 3.5 and the European average of 8.2, being the fifth country with the lowest rate of nurses^20^ . Studies conducted in Europe^26^ and China^27^ show that hospitals with low nurse/patient ratios obtain better results on patients and professionals, suggesting that satisfaction with nursing care is an important index to predict patient satisfaction.

Quality management systems and accreditation of institutions are usually linked to the satisfaction of users and quality of care. On the one hand, studies^28^ argue a strong relationship between satisfaction with safety measures, reducing complications such as pressure ulcer and infections. In contrast, a study in 73 hospitals in Germany^29^ , with more than 37,000 patients, concluded that accreditation is not linked to a better quality of care as noticed by the patient. Hospital accreditation represents a step towards total quality management but may not be a key factor for the care quality.

A study carried out in 27 European Union member states between 2009-2013^30^ on quality and safety perception shows that the changes introduced in health systems during the recession have raised concerns about safety among the population. Despite increasing global spending, European citizens are increasingly concerned about their experience within the health system. Patient satisfaction continues a pending issue in the quality of care management systems.

The results should be carefully considered. It is an ecological study that uses aggregate data in its analysis, being possible the presence of biases, such as the ecological fallacy. However, these studies are widely used designs to study the trend of indicators. Other variables such as age, educational level or level of income that are considered social determinants of health and that can influence the level of satisfaction have not been considered. On the other hand, the results of this time series can be influenced by an economic crisis that has meant an important disinvestment in the health system, with repercussions on the population’s health^31^ . However, the number of years included is limited to assess the possible impact that recent events such as the economic crisis have had on users’ satisfaction. Despite this, the results are important since they show that indicators of user satisfaction can be considered valid tracers to monitor changes, along with other outcome indicators such as mortality.

In conclusion, a static tendency was observed in the perception of users about how the public health system works. The decreasing trend in the percentage of patients who consider that the assistance has improved in primary care, specialized care and hospitalization during the observed period was highlighted. Regarding the satisfaction with medical and nursing professionals, the trend was static. The only indicator that showed a growing trend was the degree of satisfaction with knowledge and follow-up of health problems by the specialist physician and pediatrician. No large differences were observed in the trends of the indicators studied in the autonomous communities.

Factors such as investment in health, human resources, quality management systems and accreditation, added to the heterogeneity in the autonomous communities, can influence the satisfaction indicators of the Spanish national health system. Research with more complex than ecological designs is necessary to determine the impact of these factors on user satisfaction. The challenge is to achieve a better understanding of underlying factors that cause differences in satisfaction, to focus improvement strategies in areas of dissatisfaction where the needs and patients’ expectations are not covered.
