# Supplementary material for: User satisfaction in the spanish health system: trend analysis
Source: Rev Saude Publica. 2019 Sep 18;53:87. doi: 10.11606/S1518-8787.2019053001506 (PMC6763284; doi:10.11606/S1518-8787.2019053001506)
Supplement: Translation

# Translation: Satisfacción de los usuarios en el sistema de salud español: análisis de tendencias

**Authors:** Víctor Pérez-Cantó, Loreto Maciá-Soler, Víctor M González-Chordá

**Affiliations:** I Universidad de Alicante. Departamento de Enfermería. Alicante, AL, España; II Univesidad de Alicante. Unidad Científica de Innovación Empresarial “Ars Innovatio”. Alicante, AL, España; III Universitat Jaume I. Departamento de Enfermería. Castellón, CS, España

**Keywords:** Satisfacción del Paciente, Sistemas Nacionales de Salud, Calidad de la Atención de Salud, Investigación sobre Servicios de Salud

## Abstract

**OBJETIVO:**

Analizar la tendencia de indicadores de opinión y satisfacción del sistema nacional de salud español de 2005 a 2017.

**MÉTODOS:**

Estudio ecológico de series temporales analizando la tendencia de ocho indicadores de opinión y satisfacción sobre el sistema nacional de salud y sus comunidades autónomas de 2005 a 2017. Los datos se obtuvieron del Ministerio de Sanidad, Servicios Sociales e Igualdad y del Barómetro Sanitario. Se utilizó el método de autoregresión de Prais-Winsten.

**RESULTADOS:**

Se observó una tendencia estática en la percepción de los usuarios sobre el funcionamiento del sistema sanitario (APC = 1,898; IC95% -0,954–4,751) y decreciente sobre la opinión en la mejora de la atención primaria (APC = -0.283; IC95% -0,335– -0.121), especializada (APC = -0,241; IC95% -0.74– -0.109) y hospitalización (APC = -0.171; IC95% -0,307– -0,036). La satisfacción con el conocimiento y seguimiento por el médico de familia y pediatra mostró una tendencia creciente (APC = 7,939; IC95% 3,965–11,914). La satisfacción con los profesionales de medicina y enfermería fue estática. No se observaron grandes diferencias en las tendencias de los indicadores estudiados en las comunidades autónomas.

**CONCLUSIONES:**

Se observó una tendencia negativa en la opinión de los usuarios del sistema nacional de salud español. Financiación, recursos humanos, sistemas de gestión de calidad y diferencias en las comunidades autónomas pueden ser algunas de las causas.

## INTRODUCCIÓN

Los sistemas sanitarios pretenden mejorar la salud de los ciudadanos mediante la curación, prevención y rehabilitación. Sin embargo, se ven influidos por factores políticos, sociales, culturales y económicos de cada país. Los sistemas de gestión de la calidad permiten asegurar la calidad intrínseca y percibida de las prestaciones. La calidad intrínseca se centra en el diseño, ejecución y evaluación de procesos. La calidad percibida está dirigida a la evaluación y satisfacción de los usuarios^1^ .

La satisfacción, entendida como la capacidad de generar una experiencia positiva de los usuarios y la población en contacto con los servicios sanitarios, ha sido ampliamente estudiada desde los años 60, a pesar de su naturaleza subjetiva^2^ . Gobiernos y analistas de políticas han utilizado la satisfacción del paciente como un enfoque para evaluar el desempeño de los sistemas sanitarios. Las instituciones regularmente monitorizan la satisfacción de sus pacientes y desarrollan estrategias para mejorar la calidad y lograr una mejor posición en el mercado. Conocer la satisfacción permite predecir, entre otros, el cumplimiento terapéutico y el posible retorno ante un nuevo episodio. Estos dos puntos son estratégicos y su resultado influye directamente sobre los costes, rentabilidad y sostenibilidad de las organizaciones^3^ .

La evaluación de la satisfacción de usuarios no está exenta de problemas metodológicos. La satisfacción se ve influida por características de los pacientes como edad, sexo, estado civil, educación, nivel de ingresos o estado de salud^4 , 5^ . Las personas de mayor edad suelen estar más satisfechas y la insatisfacción aumenta conforme se percibe un grado de salud peor^6^ . Además, se utilizan comúnmente instrumentos de medida de elaboración propia, validados en pocos casos^7^ .

Se debe considerar también la influencia de factores socioeconómicos. La reciente crisis económica en Europa ha ejercido una gran presión sobre los sistemas de salud. Algunos países tomaron medidas restrictivas como reducción de la cartera de servicios, gasto en medicación o recortes en personal y salarios. Estas medidas pueden incrementar desigualdades sociales en la cobertura asistencial y repercutir negativamente en la seguridad y satisfacción de los usuarios^8^ .

Existen vías para controlar el gasto y mejorar la rentabilidad basadas en la implantación de sistemas de gestión de la calidad y la mejora de resultados de la atención, por ejemplo, sobre mortalidad hospitalaria, reducción de estancias, readmisiones o satisfacción del paciente, entre otras. Por lo tanto, la satisfacción del paciente se ha establecido como un indicador clave de resultado para evaluar la calidad y eficiencia de los servicios sanitarios, coincidiendo con las líneas estratégicas de la Organización Mundial de la Salud (OMS)^9^ .

El sistema nacional de salud se pone en marcha en España en 1986, tras la promulgación de la Ley General de Sanidad^10^ . Algunas características principales son el derecho de todo ciudadano a la salud, financiación de carácter público y provisión de servicios que garanticen la calidad de la asistencia, en un sistema sanitario descentralizado en comunidades autónomas.

El proceso de descentralización de competencias finalizó en 2001. En 2003, se promulgó la Ley de Cohesión y Calidad del Sistema Nacional de Salud para garantizar la participación ciudadana, calidad y la equidad de la asistencia en el territorio nacional. Esta ley establece los principios fundamentales sobre calidad asistencial y el desarrollo de herramientas como la cartera de servicios, formación y desarrollo de los profesionales, investigación, historia clínica digital única, uso de guías, protocolos e indicadores, o un sistema de información único^11^ . Sin embargo, no especifica la necesidad establecer un sistema de gestión de la calidad para todo el territorio nacional o en sus comunidades autónomas. Tras 15 años desde su implantación, algunos de los principales problemas son la falta de equidad en gasto y financiación, nivel de salud de los ciudadanos, accesibilidad a los servicios sanitarios y utilización de recursos^12^ .

Aportar información relacionada con calidad es un aspecto de interés para la salud pública. La mejora de los sistemas sanitarios y, en este sentido, examinar tendencias en la satisfacción de los usuarios, puede permitir evaluar el impacto de las políticas sanitarias adoptadas, observando la influencia de estos cambios sobre las opiniones, experiencias y actitudes de los ciudadanos en materia de salud^13^ . Los estudios de tendencias en España son limitados y no hay análisis de tendencias de indicadores relacionados con la satisfacción de los usuarios del sistema sanitario.

El objetivo de este estudio fue analizar la tendencia de los indicadores de opinión y satisfacción del Sistema Nacional de Salud español desde el 2005 el 2017.

## MÉTODOS

Estudio ecológico de series temporales que analizó la tendencia de la satisfacción de los usuarios del sistema nacional de salud español, y sus comunidades autónomas, entre 2005 y 2017.

Las variables fueron la satisfacción con el funcionamiento del sistema sanitario, con la información y con los cuidados y la atención recibida, además de la mejora percibida por los usuarios. Para ello, se analizó la tendencia de ocho indicadores de opinión y satisfacción de los usuarios sobre el sistema nacional de salud español y sus comunidades autónomas. Tres indicadores se obtuvieron de la serie de Indicadores Clave del Sistema Nacional de Salud (SNS) y de sus comunidades autónomas, a través del Sistema de Información Sanitaria del Ministerio de Sanidad, Servicios Sociales e Igualdad^14^ , y cinco indicadores del Barómetro Sanitario^15^ ( Cuadro ).

**Cuadro:** Indicadores de satisfacción seleccionados para realizar el estudio. España, 2018.

Indicador	Fórmula/pregunta
Indicadores Clave	

Satisfacción con el funcionamiento del SNS^a^	Media de las valoraciones del grado de satisfacción recogidas en una escala *likert* de 1 (“muy insatisfecho”) a 10 (“totalmente satisfecho”).
Satisfacción con el conocimiento del historial y el seguimiento de sus problemas de salud por el médico de familia y el pediatra^b^	
Satisfacción con la información recibida en la consulta del médico especialista^a^	

Barómetro sanitario	

Satisfacción con los cuidados y atención por parte del personal médico^c^	Media de las valoraciones del grado de satisfacción recogidas en una escala *likert* de 1 (“totalmente insatisfactorio)” a 10 (“totalmente satisfactorio”).
Satisfacción con los cuidados y atención por parte del personal de Enfermería^c^	
Porcentaje de encuestados que creen que la atención primaria ha mejorado^c^	En su opinión, ¿cada uno de los siguientes servicios asistenciales de la sanidad ha mejorado, ha empeorado o sigue igual respecto a los últimos cinco años?
Porcentaje de encuestados que creen que las consultas de atención especializada han mejorado^c^	
Porcentaje de encuestados que creen que la hospitalización ha mejorado^c^	

SNS: Sistema Nacional de Salud

^a^Último año de datos disponibles: 2017.

^b^Último año de datos disponibles: 2013.

^c^Último año de datos disponibles: 2016.

La serie de Indicadores Clave permite observar cambios y tendencias desde 1990. Actualmente, la lista completa asciende a 247 indicadores (accesibilidad, estado de salud, recursos, gasto, seguridad y satisfacción, entre otros). Se estudian indicadores con definiciones estandarizadas y de uso internacional, que son accesibles para su consulta y análisis en un único repositorio. Estos indicadores se calculan obteniendo los datos de diferentes fuentes con una amplia cobertura poblacional, como el Conjunto Mínimo Básico de Datos de altas hospitalarias, el Sistema de Información de Atención Primaria, estadísticas intercensales del Instituto Nacional de Estadística o la Encuesta Nacional de Salud, entre otras^14^ .

El Barómetro Sanitario es un estudio de opinión, con periodicidad anual, realizado desde 1993 por el mismo ministerio en colaboración con el Centro de Investigaciones Sociológicas español. Está diseñado para conocer el grado de satisfacción ciudadana con los servicios sanitarios públicos, el impacto de las medidas vinculadas a políticas sanitarias, el nivel de conocimiento de los ciudadanos y la opinión pública sobre estas políticas. La información se recoge mediante cuestionarios, con un diseño muestral polietápico, estratificado por conglomerados, con selección aleatoria de las unidades de muestreo, y un total de 7.800 encuestas anuales^15^ .

De acuerdo con las definiciones de los indicadores en el Sistema de Información del SNS (Indicador Clave) o el Barómetro Sanitario, los indicadores incluidos en este estudio se estiman mediante medias, sumando las valoraciones del grado de satisfacción, dividido por número de individuos encuestados, exceptuando los tres últimos indicadores que se estiman como porcentajes.

Se incluyeron datos desde 2005, ya que en años anteriores las series estaban interrumpidas y no había datos disponibles en los indicadores analizados. El último año fue 2017, excepto algún indicador en el que la serie histórica finalizaba en 2013 o 2016 por la disponibilidad de los datos del sistema de información sanitaria. Se excluyeron las ciudades autónomas Ceuta y Melilla, ya que no se disponía de los completos de toda la serie histórica.

La estimación de las tendencias se basó en el cálculo del cambio porcentual anual (Annual Percent Change – APC) y sus intervalos al 95% de confianza (IC95%). Se utilizó el método de Prais-Wisten de autorregresión para el análisis de series temporales de datos cuantitativos. Así, se obtuvo una tendencia global para un determinado período que se determinó como creciente (el IC95% es positivo y no incluye el valor 0) estática (el IC95% incluye el valor 0) o decreciente (el IC95% es negativo y no incluye el valor 0). El análisis se realizó utilizando el programa Stata 14.0.

El estudio se llevó a cabo con datos agregados que se obtienen de fuentes públicas, siendo accesibles en abierto para su consulta y explotación. Por este motivo, no se solicitó la aprobación del estudio por parte de un comité de ética e investigación.

## RESULTADOS

El grado de satisfacción con el funcionamiento del sistema sanitario aumentó de un 6,25 en 2005 a 6,68 en 2017, mostrando una tendencia estática en todas las comunidades y en el conjunto de España, excepto Madrid, que mostró una tendencia creciente (APC = 7,451; IC95% 0,433–14.470). Asturias fue la única que mostró una variación anual negativa, aunque no fue significativa ( Tabla 1 ). La información recibida en la consulta del médico especialista fue estática en el conjunto de España (APC = 2,585; IC95% -2,078–2,085) y en las comunidades autónomas excepto en Canarias con una tendencia creciente significativa (APC = 0,942; IC95% -0,200–6,337). Se apreció una variación anual negativa no significativa en Baleares, Asturias, Rioja, Extremadura, y Cataluña.

**Tabla 1:** Grado de satisfacción con el funcionamiento del sistema sanitario público. España, 2018.

CCAA	2005	2017	APC	IC95%	Tendencia
Andalucía	6,14	6,27	1,031	-4,239–6,302	Estática
Aragón	6,41	7,29	2,210	-2,114–6,536	Estática
Asturias	7,26	7,22	-0,239	-3,338–2,859	Estática
Baleares	5,83	6,98	0,845	-2,560–4,252	Estática
Canarias	5,37	6,10	1,014	-1,749–3,778	Estática
Cantabria	6,33	7,35	1,542	-1,884–4,969	Estática
Castilla y León	6,40	7,07	0,735	-1,677–3,148	Estática
Cataluña	5,96	6,51	1,012	-3,033–5,058	Estática
Extremadura	6,30	6,61	0,726	-2,176–3,629	Estática
Galicia	5,56	6,49	2,302	-0,922–5,527	Estática
La Mancha	6,61	6,65	0,388	-2,036–2,813	Estática
Madrid	6,10	6,82	7,451	0,433–14,470	Creciente
Murcia	6,01	7,10	1,475	-1,455–4,405	Estática
Navarra	6,71	7,06	1,246	-2,453–4,946	Estática
País Vasco	6,51	7,33	1,265	-2,348–4,879	Estática
Rioja	6,56	6,99	0,090	-4,924–5,106	Estática
Valencia	6,12	6,71	1,852	-2,340–6,045	Estática
España	6,25	6,68	1,898	-0,954–4,751	Estática

CCAA: comunidades autónomas; APC: annual percent change

La tendencia en la satisfacción con el conocimiento del historial y seguimiento por el médico de familia y pediatra fue creciente en el conjunto de España (APC = 7,939; IC95% 3,965–11,914) aumentando 0,6 puntos en el período. Madrid, Andalucía, Navarra, Canarias y Galicia mostraron una tendencia creciente significativa. En el resto de las comunidades autónomas, la tendencia fue estática. Las comunidades que mostraron una variación anual decreciente fueron, Baleares y Asturias ( Tabla 2 ).

**Tabla 2:** Grado de satisfacción con la información recibida en la consulta del médico especialista y médico de familia sobre su salud. España, 2018.

CCAA		2005	2017*	APC	IC95%	Tendencia
Andalucía	Especialista	7,26	7,12	0,364	-5,344–5,250	Estática
	Médico	6,97	7,59	6,183	2,799–9,567	Creciente
Aragón	Especialista	7,16	7,71	1,248	-3,314–3,403	Estática
	Médico	7,82	8,18	1,441	-1,953–4,835	Estática
Asturias	Especialista	8,02	7,24	-0,881	-1,622–4,118	Estática
	Médico	7,44	7,31	-0,034	-2,638–2,568	Estática
Baleares	Especialista	7,32	6,8	-2,711	-4,044–4,773	Estática
	Médico	7,00	7,29	-0,702	-3,226–1,821	Estática
Canarias	Especialista	6,32	6,84	0,942	0,200–6,337	Creciente
	Médico	6,87	7,42	5,202	2,236–8,168	Creciente
Cantabria	Especialista	7,22	7,65	1,479	-3,484–4,951	Estática
	Médico	7,63	7,76	0,555	-3,261–4,372	Estática
Castilla y León	Especialista	7,12	7,35	0,423	-2,745–6,207	Estática
	Médico	7,24	7,65	1,504	-3,262–6,272	Estática
Cataluña	Especialista	6,87	6,95	-0,046	-2,255–5,214	Estática
	Médico	6,67	7,4	2,968	-2,179–8,117	Estática
Extremadura	Especialista	7,59	7,56	-0,273	-6,633–1,210	Estática
	Médico	7,53	7,46	2,044	-4,223–8,313	Estática
Galicia	Especialista	6,08	7,14	2,879	-1,463–1,144	Estática
	Médico	6,46	7,33	4,063	0,837–7,289	Creciente
La Mancha	Especialista	7,17	7,48	0,888	-2,465–4,192	Estática
	Médico	7,30	7,96	3,199	-0,920–7,319	Estática
Madrid	Especialista	6,68	7,08	0,863	-0,945–6,705	Estática
	Médico	7,03	7,47	8,959	5,640–12,279	Creciente
Murcia	Especialista	7,06	8,07	3,268	-4,695–9,865	Estática
	Médico	7,15	7,62	1,094	-3,678–5,868	Estática
Navarra	Especialista	7,09	7,42	0,044	-2,796–4,572	Estática
	Médico	7,00	8,29	5,771	4,948–6,594	Creciente
País Vasco	Especialista	6,86	7,21	0,733	-5,287–4,740	Estática
	Médico	7,03	7,55	0,587	-2,971–4,146	Estática
Rioja	Especialista	7,64	7,2	-0,477	-3,507–1,744	Estática
	Médico	7,54	8,04	1,278	-1,923–4,481	Estática
Valencia	Especialista	6,57	7,26	1,730	-3,150–2,195	Estática
	Médico	6,67	7,46	2,637	-1,903–7,178	Estática
España	Especialista	6,91	7,19	2,585	-2,078–2,085	Estática
	Médico	6,96	7,56	7,939	3,965–11,914	Creciente

CCAA: comunidades autónomas; APC: annual percent change

* Los datos relacionados con la satisfacción del médico de familia son de 2013.

La satisfacción de los usuarios con el personal de enfermería mostró una tendencia estática en el conjunto de España (APC = 1,898; IC95% -0,954–4,751) y en todas las comunidades autónomas. Del mismo modo, la atención por parte del personal médico se mostró estática en su conjunto (APC = 3,227; IC95% -7,076–13,531) al igual que todas las comunidades autónomas, excepto Asturias que mostró una tendencia decreciente (APC = -10,486; IC95% -12,798– -8,174) con un descenso de más de 10 puntos ( Tabla 3 ).

**Tabla 3:** Grado de satisfacción con la atención recibida por los profesionales de enfermería y medicina. España. 2018.

CCAA		2005	2016	APC	IC95%	Tendencia
Andalucía	Enfermería	7,51	7,28	0,108	-4,407–4,625	Estática
	Medicina	7,46	7,15	-0,110	-4,374–4,153	Estática
Aragón	Enfermería	7,85	8,16	1,563	-3,377–6,504	Estática
	Medicina	7,86	8,25	1,250	-2,501–5,002	Estática
Asturias	Enfermería	8,25	7,49	-1,069	-3,682–1,543	Estática
	Medicina	8,19	7,36	-10,486	-12,798– -8,174	Decreciente
Baleares	Enfermería	7,29	7,82	0,036	-3,197–3,270	Estática
	Medicina	7,15	7,52	-0,296	-3,447–2,853	Estática
Canarias	Enfermería	6,90	7,57	0,771	-3,325–4,868	Estática
	Medicina	6,70	7,36	1,162	-3,327–5,652	Estática
Cantabria	Enfermería	7,60	8,04	1,690	-4,130–7,510	Estática
	Medicina	7,61	7,95	0,857	-3,040–4,755	Estática
Castilla y León	Enfermería	7,08	7,40	1,939	-3,715–7,593	Estática
	Medicina	7,47	7,55	1,380	-4,459–7,219	Estática
Cataluña	Enfermería	7,39	7,46	1,219	-4,562–7,001	Estática
	Medicina	7,24	7,23	0,288	-4,814–5,391	Estática
Extremadura	Enfermería	7,66	7,53	0,415	-3,552–4,383	Estática
	Medicina	7,60	7,53	0,151	-3,989–4,291	Estática
Galicia	Enfermería	6,39	7,28	1,279	-1,939–4,499	Estática
	Medicina	6,28	7,13	1,252	-2,262–4,768	Estática
La Mancha	Enfermería	7,56	7,68	0,142	-3,511–3,797	Estática
	Medicina	7,18	7,22	-0,306	-3,996–3,384	Estática
Madrid	Enfermería	7,14	7,58	2,342	-3,964–8,650	Estática
	Medicina	6,98	7,48	1,251	-3,216–5,719	Estática
Murcia	Enfermería	6,96	7,47	1,505	-2,687–5,698	Estática
	Medicina	6,92	7,66	2,699	-1,658–7,058	Estática
Navarra	Enfermería	7,67	8,00	0,816	-2,933–4,565	Estática
	Medicina	7,55	7,85	0,625	-2,668–3,918	Estática
País Vasco	Enfermería	7,36	7,87	4,188	-1,835–10,218	Estática
	Medicina	7,14	7,65	3,531	-2,631–9,694	Estática
Rioja	Enfermería	8,16	7,75	-0,458	-3,543–2,626	Estática
	Medicina	7,93	7,75	0,006	-3,867–3,879	Estática
Valencia	Enfermería	7,02	7,20	0,654	-5,079–6,389	Estática
	Medicina	6,96	7,25	1,127	-4,766–7,021	Estática
España	Enfermería	7,30	7,48	1,898	-0,954–4,751	Estática
	Medicina	7,20	7,37	3,227	-7,076–13,531	Estática

CCAA: comunidades autónomas; APC: annual percent change

Se observó una tendencia decreciente en la mejora percibida por los usuarios en los servicios de atención primaria en el conjunto de España (APC = -0,238; IC95% -0,335– -0,121), así como en cinco comunidades (Asturias, Cantabria, Andalucía, Cataluña y Madrid). El porcentaje de usuarios que creen que la atención en consultas especializadas ha mejorado mostró una tendencia decreciente en España (APC = -0,241; IC95% -0,74– -0,109) y cuatro comunidades (País Vasco, Asturias, Andalucía y Cantabria); las demás se mostraron estáticas. Del mismo modo, se observó una tendencia decreciente en los servicios de hospitalización en España (APC = -0,171; IC95% -0,307– -0,036) y en tres comunidades autónomas (Murcia, Asturias y Andalucía). Ninguna comunidad mostró una tendencia creciente en el porcentaje de encuestados que valoraron si la atención en estos tres servicios había mejorado en los últimos cinco años.

## DISCUSIÓN

De forma general, hube una tendencia estática en España y la mayoría de sus comunidades autónomas en los indicadores: grado de satisfacción con el funcionamiento del sistema sanitario público, información recibida por el médico especialista y conocimiento del historial además de seguimiento por parte del médico de familia y pediatra. Hay pocos estudios de tendencias en satisfacción a nivel nacional e internacional y es escasa la investigación que vincula tendencias a mejoras realizadas en base a la satisfacción del paciente^16^ . Sin embargo, los casos con tendencia creciente sobre los indicadores vinculados al desarrollo de la historia clínica digital del SNS en España se relacionan con mejoras identificadas por los usuarios cuando acuden a un centro sanitario respecto a la información sobre su enfermedad y el orden en la documentación^17^ .

Analistas argumentan que una mayor financiación no conduce necesariamente a una mejor satisfacción y no pueden explicar una gran parte de la diferencia en las calificaciones de satisfacción entre países^18 , 19^ . Un estudio realizado en Francia^18^ con más de 10.000 pacientes mostró que los pacientes quedaron satisfechos con su estancia en el hospital a pesar de las reducciones en el gasto hospitalario. En España, un análisis de tendencias sobre indicadores de asistencia especializada^19^ mostró un aumento de 9,5 puntos en el gasto, con una tendencia decreciente en la satisfacción de los usuarios de dichos servicios, con peores resultados en salud (aumento de infecciones y mortalidad intrahospitalaria).

El último informe anual del sistema nacional de salud español^20^ muestra como algunas comunidades autónomas con elevado gasto sanitario (País Vasco, Extremadura, Navarra, Murcia, Asturias y Cantabria) presentaron valoraciones positivas de los usuarios en la mayoría de los indicadores, mientras que Baleares y Andalucía mostraron peores resultados en la mayoría de los indicadores, coincidiendo con un gasto sanitario menor. La heterogeneidad de las comunidades autónomas en financiación puede influir en los resultados de opinión de los usuarios. Además, se observó un gradiente norte-sur, ya que los mejores resultados en los indicadores estudiados fueron en la mitad norte del país y los peores en el sur. Dicho gradiente ya ha sido mencionado en investigaciones previas^21^ relacionado con el descenso del gasto público en salud y un aumento de las desigualdades entre comunidades autónomas.

Se observó tendencia decreciente en el porcentaje de pacientes que creen que la atención primaria, consultas de atención especializada y hospitalización, tres pilares del sistema de salud, han mejorado. Los resultados en hospitalización coinciden con otros estudios de tendencias en Francia^18^ y Alemania^22^ . Eso sugiere que aspectos relacionados con la calidad como información, comodidad y agilidad de los sistemas influyen cada vez más en la satisfacción de los usuarios^23^ .Los principales problemas en la atención primaria pueden ser la infrafinanciación y la heterogeneidad causada por la descentralización de las competencias^24^ . Sin embargo, se deben reflexionar bien sobre las posibles estrategias a implantar. Un estudio realizado en Finlandia entre 1998 y 2011 mostró que, a pesar de las reformas gubernamentales, los pacientes estaban menos satisfechos con la atención primaria, sobre todo con la accesibilidad y la continuidad de los cuidados^25^ .

Por otra parte, la satisfacción con los profesionales de enfermería y médicos en el conjunto de España mostró una tendencia estática, a pesar de la tendencia creciente de profesionales de enfermería y medicina en los últimos años^19^ . Las comunidades autónomas con un alto grado de satisfacción en relación a los profesionales de enfermería y medicina presentaron las tasas más altas en dotación de profesionales^20^ , observándose el mismo gradiente norte-sur. La influencia de la tasa de profesionales en la calidad de la atención y satisfacción es tratada por diversos autores, aunque no se han identificado estudios a nivel nacional vinculados a medicina o enfermería.

España dispone de una tasa de médicos de 3,8 para cada mil habitantes en su SNS (la media europea es de 3,5), ocupando el séptimo puesto con más médicos en la Europa. La tasa de enfermeras en España es de 3,5 y la media europea de 8,2, siendo el quinto país con menor tasa de enfermeras^20^ . Estudios realizados en Europa^26^ y China^27^ muestran que hospitales con ratios enfermera/paciente bajos obtienen mejores resultados sobre pacientes y profesionales, sugiriendo que la satisfacción con la atención de enfermería es un índice importante para predecir la satisfacción del paciente.

Los sistemas de gestión de la calidad y la acreditación de las instituciones normalmente se vinculan a satisfacción de los usuarios y calidad de la atención. Por un lado, estudios^28^ argumentan una relación sólida entre satisfacción con medidas de seguridad disminuyendo las complicaciones como úlcera por decúbito e infecciones. En contraposición, un estudio en 73 hospitales en Alemania^29^ , con más de 37.000 pacientes, concluyó que la acreditación no está vinculada a una mejor calidad de la atención según lo percibido por el paciente. La acreditación hospitalaria representa un paso hacia la gestión de la calidad total, pero puede no ser un factor clave para la calidad de la atención.

Estudio realizado en 27 estados miembros de la Unión Europea entre 2009–2013^30^ , sobre la percepción de calidad y seguridad, pone de manifiesto que los cambios introducidos en los sistemas de salud durante la recesión han aumentado las preocupaciones sobre la seguridad entre la población. A pesar del gasto global creciente, los ciudadanos europeos están cada vez más preocupados sobre su experiencia dentro del sistema de salud. La satisfacción de pacientes continúa una asignatura pendiente en los sistemas de gestión de la calidad asistencial.

Los resultados deben considerarse con cautela. Se trata de un estudio ecológico y utiliza datos agregados en su análisis, siendo posible la presencia de sesgos como la falacia ecológica. Sin embargo, dichos estudios son diseños utilizados ampliamente para estudiar la tendencia de indicadores. No se ha contemplado otras variables como la edad, nivel educativo o nivel de ingresos que se consideran determinantes sociales de la salud y que pueden influir en el nivel de satisfacción. Por otra parte, los resultados de esta serie temporal pueden verse influidos por una crisis económica que ha supuesto una importante desinversión en el sistema sanitario, con repercusiones sobre la salud de la población^31^ . Por otro lado, el número de años incluidos es limitado para valorar el posible impacto que hechos recientes como la crisis económica han podido tener en la satisfacción de los usuarios. A pesar de ello, los resultados son de interés ya que evidencian que los indicadores de satisfacción de los usuarios se pueden considerar trazadores válidos para monitorizar cambios, junto con otros indicadores de resultado como la mortalidad.

En conclusión, se observó una tendencia estática en la percepción de los usuarios sobre el funcionamiento del sistema sanitario público. Destacó la tendencia decreciente en el porcentaje de pacientes que consideran que la asistencia ha mejorado en atención primaria, especializada y hospitalización durante el periodo observado. Con respecto a la satisfacción con los profesionales de medicina y enfermería, la tendencia fue estática. El único indicador que mostró una tendencia creciente fue el grado de satisfacción con el conocimiento y seguimiento de los problemas de salud por el médico especialista y pediatra. No se observaron grandes diferencias en las tendencias de los indicadores estudiados en las comunidades autónomas.

Factores como inversión en salud, recursos humanos, sistemas de gestión de calidad y acreditación, sumados a la heterogeneidad en las comunidades autónomas, pueden influir en los indicadores de satisfacción del sistema nacional de salud español. Son necesarias investigaciones con diseños más complejos que los ecológicos para determinar el impacto de estos factores en la satisfacción de los usuarios. El desafío es lograr una mejor comprensión de factores subyacentes que causan diferencias en la satisfacción, para enfocar estrategias de mejora en áreas de insatisfacción en las que las necesidades y expectativas de los pacientes no estén cubiertas.
